# Directivity and noise reduction in hearing aids: speech perception and benefit

**DOI:** 10.1590/S1808-86942010000500016

**Published:** 2015-10-22

**Authors:** Camila Angélica Quintino, Maria Fernanda Capoani Garcia Mondelli, Déborah Viviane Ferrari

**Affiliations:** 1 MSc - University of São Paulo - Product Manager for a hearing aid company; 2 PhD in Communication Disorders - Hospital de Reabilitação de Anomalias Craniofaciais HRAC/USP. Pofessor - Department of Speech and Hearing Therapy - Dentistry School - FOB/US P; 3 PhD in Psychology - Neurosciences and Behavior - USP/SP. Professor - Department of Speech and Hearing Therapy - Dentistry School - FOB/USP Centro de Pesquisas Audiológicas (CPA) do Hospital de Reabilitação de Anomalias Craniofaciais (HRAC) - USP/Bauru e Faculdade de Odontologia de Bauru FOB/USP

**Keywords:** hearing aid, perception, hearing loss.

## Abstract

**Abstract:**

Hearing aid.

**Aim:**

To compare the performance, benefit and satisfaction of users of ITE, CIC and BTE digital hearing aid with noise reduction and omnidirectional and directional microphones.

**Method:**

34 users of hearing aid were evaluated by means of speech perception in noise tests and APHAB and IOI self assessment questionnaires. Prospective study.

**Results:**

Better results were obtained by users of ITE, CIC and directional hearing aids, however, no statistical significance was found between the groups.

**Conclusion:**

Directivity improved speech perception in noise and benefit in daily life situations.

## INTRODUCTION

One of the greatest difficulties of the hearing impaired is to understand speech, especially in the presence of competitive noise^1^, ^2^, ^3^, ^4^. The speech masking potential by competitive noise is expressed by the signal/noise (S/N) ratio, which is the difference between the intensity of a signal (speech) and the intensity of a competitive sound (noise), when presented simultaneously. The lower this association, the greater is the loss in speech understanding, both for normal hearing as well as for hard of hearing individuals^5^.

New circuits present in noise reduction algorithms and directional microphones have been introduced to cochlear implants^6^ and digital personal sound amplification devices (PSAD) with the intent of improving user performance^7^. Noise reduction algorithms aim at improving speech intelligibility, enhance comfort and reduce the hearing stress of PSAD users when in situations in which the S/N ratio is not favorable^8^,^9^.

The underlying logic of each of these algorithms varies according to the manufacturer. In most of the devices, the digital processor does the statistical analysis of the input signal, in each one of the PSAD frequency bands, in order to check to see which signal is predominant and to dampen band gain when noise is dominant^10^.

Studies developed with the aim of assessing noise reduction algorithms show conflicting results. Some authors find improvements in the clinical evaluation of speech in the presence of noise when noise reduction algorithms are used,^11^, ^12^, ^13^,^8^ while others find improvements only on self-assessment questionnaires^14^, ^15^, ^16^.

Another way to improve speech perception in the presence of noise is to use directional microphones, which have different sensitiveness according to the sound wave incidence angle. These microphones are usually more sensitive to the sounds which come from the front of the user. Considering that the sound signals of greater interest come from the front and those of less interest come from behind, PSAD directional microphones have a significant potential concerning improvements in the S/N ratio^2^,^17^,^18^. We carried out a study with 10 normal hearing individuals in order to check the effectiveness of the directional microphones and the noise reduction for future use in hard of hearing individuals, and we confirmed the improvement in speech understanding in noisy environments, and such results were used as reference to employ the aforementioned algorithims^19^.

The most commonly found directional microphones in PSAD are the conventional and the dual. In a conventional design, there is a single microphone with two sound inputs (anterior and posterior). These sound inputs lead the sound to two distinct cavities separated by a diaphragm. In the posterior cavity there is an acoustic network which delays the sound wave entering through this cavity, until it reaches the diaphragm. Such delay makes sound waves from both inputs reach the diaphragm at the same time, preventing it from moving, thus canceling the sound wave that comes from behind^20^.

In a dual design, two omnidirectional identical microphones (anterior and posterior) are used, connected by an electrical network. In this case, the user can toggle between the directional or omnidirectional modes.

Researchers^1^,^16^,^20^,^21^ noticed an improvement in speech recognition in noise when directional microphones were used in the PSAD in comparison to only using noise reduction algorithms.

One important condition to obtain directivity is that there must be enough distance between the different microphone openings in a conventional design, or between the different microphones in a dual design. Often times, such fact can prevent the use of directional microphones in cases in which the PSAD size, in other words the cosmetic aspect, is the most important factor to be considered. Studies indicate^22^,^23^ that cosmetics is as important as the acoustic benefit provided by the PSAD.

Miniaturizations of electronic components and batteries have enabled the manufacturing of intraaural PSADs, which components are fully inserted in the ear concha and/or the user's external acoustic meatus. Besides the cosmetic benefit, one of the main advantages of intraaural PSADs, especially the intra-canal and microcanal ones, is the use of the acoustic characteristics of the external ear, generating, among other factors, greater emphasis in the high frequency amplification and a significant improvement in directivity^24^, ^25^, ^26^, ^27^. Such factors may enhance the user's performance in noisy environments.

The goal of the present study was to compare speech perception performance in noisy environments, the benefit and satisfaction obtained by hearing impaired adult users of digital PSADs with noise reduction algorithm, concerning the omnidirectional retroauricular device, the directional retroauricular, the ominidirectional intracanal and the omnidirectional microcanal devices.

## MATERIALS AND METHODS

This study was approved by the Ethics Committee, under protocol # 074/2007. All the participants in this study consented to the disclosure of the results.

### Materials

34 individuals with ages varying between 15 and 79 years (mean of 52) with bilateral mild to severe sensorineural hearing loss participated in this study (Table 1).

**Table 1:** Mean and standard deviation of the air conduction auditory thresholds in the best ear of the individuals participating in the study (n=34).

Frequency (Hz)												
	250		500		1000		2000		3000		4000	
	x	SD	X	SD	x	SD	x	SD	X	SD	X	SD
Micro canal	27	13	28,5	10	33	10	43	11	49	9	54,5	10
Intra canal	33	12	39,5	13	46,5	13	56	8	58,5	10	64,5	10
Retro Omni	31	18	38	17	53	10	61	9	65	7	65	14
Retro Dir	34	12	41	17	42	18	48	12	56	14	62	14

All the participants had been using a digital PSAD for at least one month, in one ear only (n=9) or in both (n=25). Those using it in only one hear had symmetrical hearing loss (n=3) or asymmetrical (n=6). In the latter, the PSAD was used in the best ear.

Four groups were assessed: microcanal PSAD users, identified as CIC (n=10, three single-ear-users); intracanal PSAD who will be identified as ITC (n=10, and three single-ear-users); retroauricular (behind-the-ear) devices with omnidirectional microphone (n=9, two were monaural fittings) and users of retroauricular PSAD with directional microphone (n=5, one monaural fitting) which will be retroauricular R and L, respectively.

It is worth stressing that, although we used PSAD models with different acoustic gains, both the hearing loss level and the PSAD programming were, as far as possible, controlled among the participants in order to avoid the influence of these factors on the results.

### Procedures

**PSAD Programming:**

The PSADs used had broad three channel dynamic area compression, with a minimum compression threshold equal to 20 dB HL^28^. These devices also have a noise reduction algorithm. Broadly speaking, this algorithm makes a statistical analysis of the input signal for each filter bank (LOW, MID and HIGH), it decides whether a given signal represents speech or noise, and according to it, the gain parameters for each canal are automatically modified^11^. Moreover, the PSAD has a passive sound feedback reduction algorithm.

Microcanal (CIC), intracanal (ITC) and retroauricular models had omnidirectional microphone with a circular polar pattern, while the D retroauricular model has a directional microphone with a cardioid pattern. The directional microphone of the R PSAD has an antero-posterior relation of approximately 15 to 20 dB up to 3000 Hz^20^.

All the PSADs were programmed according to the protocol recommended by the manufacturer, by means of software. After establishing the thresholds, we ran a feedback test in order to check for the possible occurrence of feedback based on the necessary gain combination, established by the thresholds obtained with the characteristics of the ear mold selected. During fine tuning, the patients' comments were considered.

The frequency response check and maximum output obtained in the real ear was carried out by means of the probe microphone measure, with the Unity System PC Probe Mic (Siemens) equipment; for which we used the ICRA type 2PB-1F1M-N stimulus, in the intensities of 55, 65 and 85 dB SPL.

### Anteroposterior Ratio evaluation

The value of the anteroposterior relation assesses PSAD sensitivity with directional microphone for signals coming from the 0° azimuth compared to the signal coming from the 180° azimuth^29^. This measure was done in the real ear by means of the Unity System PC Probe Mic (Siemens) equipment. We obtained the response from PSAD in the outer ear (REAR), and the speaker box was positioned at 180° and, later on at 0° azimuth. The difference of these two values was considered with the anteroposterior relation. This measure was done in order to estimate PSAD directivity^27^.

### Speech Perception Evaluation

Speech perception was assessed by means of the recognition of the sentences presented during noise^30^. The presentation of sentences together with competitive noise was done by means of a portable CD player (TEAC, model PD-P30) coupled to the Unity System PC Audiometer (Siemens) equipment, in the free field, in a sound treated booth. The individuals were positioned at 1 meter distant from the speakers, which were positioned at 0° and 180 ° azimuths. For this procedure we used the strategy proposed by Levitt and Rabiner^31^, called sequential or ascending-descending strategy, which determines speech recognition thresholds, which is the necessary level of the individual to correctly recognize 50% of the sentences presented, starting from a S/N ratio equal to zero.

Initially, both speech (0° azimuth) and noise (180° azimuth) were presented at the intensity of 64 dB H. The noise was kept fixed and the speech varied at 4dB intervals until the change in response, moving on to 2dB intervals, up to the end of the list.

### Assessment of PSAD benefit and satisfaction

In order to assess the benefit attained from the PSDA use in different situations of daily life we used the Abbreviated Profile of Hearing Aid Benefit - APHAB^32^ questionnaire, which contains 24 items, scored in four subscales: communication in favorable environments (FC), communication in reverberating environments (R), communication in the presence of background noise (BN) and discomfort towards environmental sounds (D). The score of the percentages of the hearing difficulties obtained with and without the use of PSAD and the benefit obtained from the use of PSAD were calculated by means of the APHAB software for Windows V1.0c (Copyright © 1997, The University of Memphis). The benefit is associated with the percentage of problems obtained with and without PSAD.

The satisfaction with the use of PSAD was assessed by means of the International Outcome Inventory for Hearing Aids - IOI-HA^33^. This questionnaire has seven questions, each one approaching the aspects associated with daily use, benefit, limitation of activities, participation restrictions, the opinion of others and quality of life (Attachment 1). Each question has five answer options, being scored from one to five (from left to right). The maximum score obtained in this questionnaire was 35. Higher scores correspond to greater satisfaction.

### Statistical method

The results were statistically analyzed by the Kruskal Wallis test and by post hoc analysis done by the Dunn test. For all the evaluations we used the significance level below 0.05.

## RESULTS

**Graph 1:**
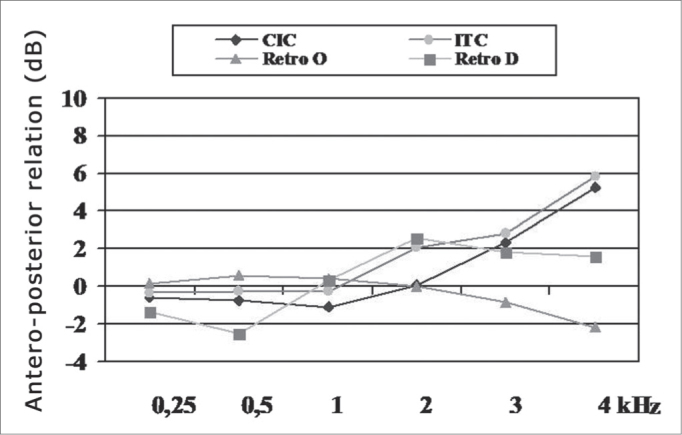
Mean values of the anteroposterior relation in the frequencies of 250 to 4,000Hz (n=34).

Graph 1 shows the mean values of the anteroposterior ratio in the frequencies of 250 to 4000Hz, for the different groups assessed.

The Kruskal Wallis test showed a statistically significant difference only in the frequencies of 3 kHz (p=0.01) and 4 kHz (p=0.00007). The Dunn test for individual comparisons showed that on frequency 3kHz there was a statistically significant difference between retroauricular with omnidirectional (O) microphone and retroauricular with directional (D) microphone groups. At the frequency of 4 kHz, we noticed a statistically significant difference between the CIC group and the O and D retroauricular groups, respectively, and between the ITC group and the other O and D retroauricular groups.

**Graph 2:**
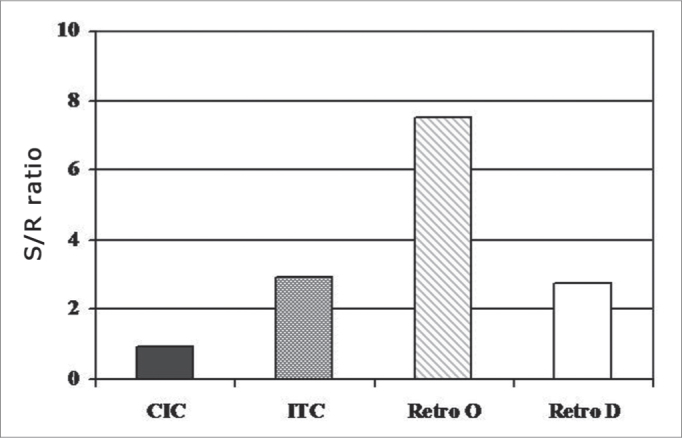
Mean values of the S/N ratio in which 50% of the sentences were recognized for the different groups evaluated (n=34).

Graph 2 shows the mean of the S/N ratio, in which 50% of the sentences were recognized, in the different groups studied.

The mean values of the S/N ratio found were 0.95, 2.95, 7.54 and 2.79 dB for groups CIC, ITC, retroauricular omnidirectional and directional, respectively. The Kruskal Wallis test did not show statistically significant differences between the groups (p=0.1068).

As far as the results obtained from the APHAB questionnaire goes, Table 2 shows the mean value of the

**Table 2:** Mean value of the percentage of problems found without the use of PSAD in the different subscales of the APHAB questionnaire, for the different groups investigated (n=34).

Evaluation situation								
		Without PSAD				With PSAD		
Groups	FC	R	RF	D	FC	R	RF	D
CIC	48,47	54,98	59,37	39,34	39,49	35,73	38,84	49,63
ITC	69,83	64,31	55,81	41,6	41,8	37,01	32,94	56,2
Retro O	59,08	60,72	65,84	53,14	23,04	34,38	24,22	71,04
Retro D	65,18	62,26	63,15	37,82	35,68	24,46	22,24	70,42

**Attachment 1.** International IOI-HA Questionnaire

1 Think of the time during which you used your personal sound amplification device in the last two weeks. For how many hours did you use your PSAD in a regular day?
  ( ) did not use ( ) less than 1 hour per day ( ) between 1 and 4 hours per day ( ) between 4 and 8 hours per day ( ) more than 8 hours per day.
2 Think about in which situation you would like to hear well, before obtaining your personal hearing amplification device. In the last two weeks, how did the personal sound amplification device help you in these situations?
  ( ) did not help at all ( ) helped a little ( ) helped moderately ( )helped much ( ) helped very much
3 Think again about the same situation in which you would like to hear well, before obtaining your Personal Sound Amplification Device. Which level of difficulty do you still find in this same situation using your sound amplification device?
  ( ) very much difficulty ( ) much difficulty ( ) moderate difficulty ( ) little difficulty ( ) no difficulty at all
4 Considering everything, do you think it is worthwhile using the personal sound amplification device? ( ) it is not worth it ( ) a little ( ) moderately ( ) it is worth using it ( ) it is very much worth using it
5 Think of the two last weeks, using the personal sound amplification device, how much did your hearing impairment affect your activities?
  ( ) very much ( ) much ( ) moderately ( ) a little ( ) not at all
6 Think about the last two weeks, using your personal sound amplification device. How much did your hearing impairment affect or upset other people?
  ( ) very much ( ) much ( ) moderately ( ) a little ( ) not at all
7 Considering everything, how do you think your personal sound amplification device changed your happiness with life or life enjoyment?
  ( ) for worse, or less life enjoyment ( ) there was no change ( ) a little more happiness in life ( ) much happiness in life ( ) very much happiness in lifepercentage of problems obtained without PSAD and with PSAD, on the different subscales for the different groups studied. The Kruskal Wallis test did not show statistically significant differences between the groups as to the percentage of problems obtained, without the use of amplification on scales FC (p=0.1426), R (p=0.8192), RF (p=0.6345) and D (p=0.6254). In regards of the percentage of problems obtained with the PSAD, also there were no statistically significant differences between the groups, on scales FC (p=0.8997), R (p=0.4321), RF (p=0.1649) and D (p=0.1389).

**Graph 3:**
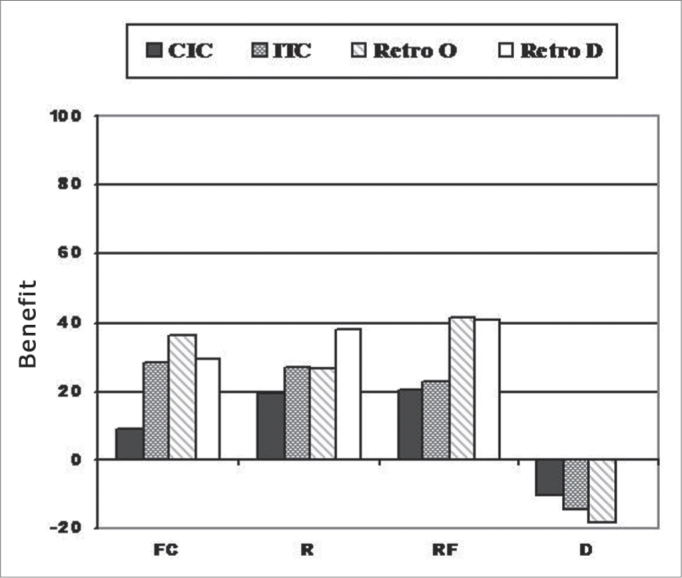
Mean values of the benefit obtained with the use of PSAD in the APHAB questionnaire for the different groups studied (n=34).

Graph 3 shows the mean values of the benefits obtained form the use of PSAD considering the different groups. The Kruskal Wallis test did not show statistically significant difference between the groups studied, on scales FC (p=0.35), R (p=0.2), RF (p=0.08) and D (p=0.2).

**Graph 4:**
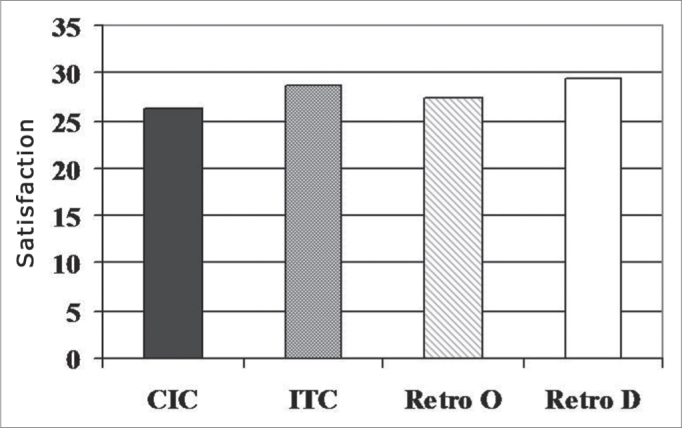
Mean values of the person's satisfaction with the use of the PSAD obtained in the IOI questionnaire for the different groups studied (n=34).

Graph 4 shows the mean values of patient satisfaction with the PSAD, obtained from the IOI questionnaire for the different groups studied. The values found for groups CIC, ITC, Retro O and D, were: 26.2; 28.8; 27.4 and 29.5, respectively. The Kruskal Wallis test did not show statistically significant differences between the groups studied (p=0.1923).

## DISCUSSION

Graph 1 shows, in the higher frequencies, significantly higher values of the anteroposterior ratio which were obtained when using the directional microphone or the intracanal or microcanal omnidirectional PSAD. These results corroborate literature findings, showing that the directional microphone has effectiveness^34^, providing sensitivity for sounds coming from the front and that the intraaural PSAD can provide greater amplification for the high frequencies because of the endaural position of the microphone as well as the shadow effect provided by the ear pinna for high frequency sounds coming from behind24,25,35.

Although we did not find statistically significant differences between the groups studied, we noticed that at frequencies 250 and 500Hz the directional microphone effect was reduced. Such result may have been the consequence of the directional microphone design of this PSAD, a consequence of implementing conventional microphones is that the total frequency response is, basically, tilted on 6 dB/octave. This means that there will be less sensitivity on the low frequency area for this type of microphone when compared to omnidirectional microphones. Wolf et al.^26^ report this characteristic of the directional microphone and stress the need to indicate it for individuals with significant hearing loss in this frequency range. Today, some circuits embed additional filters which can compensate for this change.

Researchers^36^ have also reported that the directional properties vary according to frequency. In case of low frequencies, these properties may be affected because of the head diffraction effect.

And finally, another factor which can influence directivity on low frequencies is the acoustic modification brought about by the ear molds. If one mold has ventilation, low frequency sounds and those of sufficient strong intensity coming from behind may simply pass through the ventilation without attenuation and may reach the intensity level of the original front amplified signal, thus significantly reducing directivity^37^. All users of retroauricular PSAD with directional microphone in this study have a simple invisible mold with ventilation, except for one participant who used the canal mold.

It is worth stressing that the anteroposterior ratio obtained in this study for D retroauricular PSAD in the different frequencies is lower than what has been reported by other authors^14^,^20^. This can be explained by the fact that these authors describe the anteroposterior ratio measured on the ear simulator, with the PSAD on “test” mode and with the gain in the reference position. When the PSADs are in the “test” mode, the automatic functions are off and they operate in a linear mode. In the present investigation, the measurements were all carried out in the user's ear and with the PSAD settings adjusted by the user. For this reason, these measures suffered the influence of the auricular mold's acoustics and, especially, the low PSAD compression threshold. Because of the compression threshold, a greater gain will be provided for the signals which hit the angle at which the signal amplitude will be reduced by the directional microphone than for the signals which hit from angles where there is no amplitude reduction, thus reducing the anteroposterior ratio.

There was no statistically significant difference between the groups as to the performance of speech recognition in noise (Graph 2). Such fact may have happened because of the sample number, which may have been insufficient to analyze the effects from the different devices. Moreover, the type of competitive noise used (still) may have favored the performance with the omnidirectional retroauricular PSAD. Nonetheless, even then we can see a trend towards an association between directivity of the different PSAD studied and the results obtained from the speech recognition test under noise. Graph 2 shows that the omnidirectional retroauricular PSAD requires a greater S/N ratio than the other groups in order to recognize 50% of the phrases presented. This can be explained by the polar pattern of the omnidirectional microphone of this PSAD, which has sound sensibility from all the incidence angles, and in this case, the noise processing (in 180° azimuth) may have impaired speech recognition (in 0° azimuth). These results also suggest that the noise reduction algorithm of the PSAD, working alone, may not be efficient to improve the S/N ratio for the user, matching observations from other authors^14^, ^15^, ^16^,^38^,^39^. Thus, it is important that the user be advised in relation to the real expectations when using such technology^40^.

Similar mean values of the S/N ratio were obtained for groups CIC, ITC and D retroauricular, and the lowest mean value was found for group CIC (0.95dB). Directivity given electronically and by the endaural position of the microphone starting from the middle frequencies, favored speech recognition in noisy environments, since most of the speech sound spectrum is located in this frequency range; thus justifying an improvement in the S/N ratio found in groups CIC, ITC and D retroauricular. These results are in accordance with the literature, which shows an improvement in the S/N ratio obtained from the directional microphone^9^,^14^,^21^,^38^,^41^, or by using the acoustic characteristics of the external ear^24^, ^25^, ^26^, ^27^,^42^.

In the case of the retroauricular PSAD with directional microphone, the fact that the S/N ratio obtained was higher that that of the CIC, it probably associated with the ventilation effects of the ear mold. In such case, we believe the low PSAD threshold did not influence the results because both signals (speech and noise) were given simultaneously during the test. Electroacoustic directivity studies^42^ have shown that the compression threshold does have any effect on the antero-posterior relation or on the directivity index when the signal of interest and the competitive noise are presented at the same time.

We cannot refrain from mentioning that although there were variations, as shown by the standard deviation, in average, the auditory threshold values from group CIC were lower than those from the other groups. Such fact may have influenced the speech perception results. Moreover, the individual differences on the auditory skills among the individuals assessed may have played an important role.

As to the APHAB questionnaire evaluation, there were no statistically significant differences between the groups on the different subscales assessed, both with and without PSAD. Such fact suggests that the auditory difficulties found by users in their daily lives were similar. Nonetheless, it is possible to observe that the percentage of difficulties found without the PSAD in the FC and R subscales was lower for CIC PSAD users.

As far as the benefits obtained (Graph 3) goes, all the groups had benefits on the four communication subscales we studied (FC, R, RF and), and there were no statistically significant differences between them. We stress that the average benefit with the use of CIC PSAD for communication situations in favorable (FC) and reverberating (R) situations were the lowest found. These results may also have risen from the fact that this group had lower percentage of difficulties without amplification in these subscales, when compared to the other groups.

Under situations of communication in reverberating environments, the mean value of the benefit achieved with the PSAD was found for the D retroauricular group (37.78%). In principle, such result was not expected, since many authors have reported a worse performance given by directional microphones in reverberating environments and that, depending on the distance of the sound source, the performance of these microphones will be similar to the one from the omnidirectional microphones. This happens because the reverberation makes the sound waves have a diffuse location instead of originating from one specific direction^27^,^43^. Notwithstanding, while the results from the present study are in agreement with those from Ricketts and Dhar1, who found speech improvement in reverberating environments with the use of directional (D) retroauricular PSAD when compared to the omnidirectional (O).

As to the benefits given by the PSAD in communication situations under background noise, better results were found for the O (41.61%) and D (40.91%) retroauricular PSADs. The improvement results obtained from the D retroauricular PSAD are in agreement with the speech perception results and with the literature, which reports a better performance of the directional PSAD in the presence of noise^17^,^20^,^27^,^44^. As to the O retroauricular PSAD, the results obtained from the RF subscale were similar to the ones found in other studies^11^,^14^. There is a discrepancy between the speech-perception-in-noise results obtained from the ITC, CIC and O retroauricular PSAD and the results obtained from the subjective evaluation. One of the hypotheses which could be raised is that in subjective assessment one also considers the orofacial reading effect. It is possible that the group of O retroauricular PSAD users have benefited more from the visual clues that the ITC and CIC groups.

We also observed that there were no benefits on the discomfort scale towards environmental sounds for the different groups. This finding is in agreement with the standards found for this questionnaire^32^ and it can be explained by the increase in audibility caused by the use of amplification. It is necessary to stress that the evaluation done with the probe microphone showed that in no case the maximum PSAD output was above the tolerable threshold for the users.

Regarding the evaluation of user satisfaction with the PSAD (Graph 4), there was no statistically significant difference between the groups. Nonetheless, we noticed a mild score increase for the D retroauricular PSAD group. The results obtained from this study are similar to the ones found in the IOI-HA questionnaire standards^33^ for approximately 37% of the users investigated.

Considering the results for the different aspects discussed in the IOI questionnaire, of particular importance is the fact that on question 4 (Attachment 1) retroauricular PSAD users had a lower score than the one obtained from intra-aural PSAD users. This can indicate that for the participants of the present study, the performance improvement obtained from the use of amplification overcomes cosmetic issues.

## CONCLUSION

There was no statistically significant difference in speech perception in the presence of competitive noise between the groups of digital PSAD with omnidirectional and directional noise reduction algorithms. Nonetheless, we observed that the directivity obtained by acoustic or electronic means favored speech recognition in this situation.

There were no statistically significant differences between the groups of digital PSAD with omnidirectional and directional noise reduction algorithms concerning the subjective evaluation of the benefit or user satisfaction with the amplification.
